# Zebrafish Models of Craniofacial Malformations: Interactions of Environmental Factors

**DOI:** 10.3389/fcell.2020.600926

**Published:** 2020-11-16

**Authors:** S. T. Raterman, J. R. Metz, Frank A. D. T. G. Wagener, Johannes W. Von den Hoff

**Affiliations:** ^1^Radboud Institute of Molecular Life Sciences, Nijmegen, Netherlands; ^2^Department of Dentistry-Orthodontics and Craniofacial Biology, Radboud University Medical Center, Nijmegen, Netherlands; ^3^Department of Animal Ecology and Physiology, Institute for Water and Wetland Research, Radboud University, Nijmegen, Netherlands

**Keywords:** zebrafish, craniofacial malformations, neural crest cells, environment, gene, interaction

## Abstract

The zebrafish is an appealing model organism for investigating the genetic (G) and environmental (E) factors, as well as their interactions (GxE), which contribute to craniofacial malformations. Here, we review zebrafish studies on environmental factors involved in the etiology of craniofacial malformations in humans including maternal smoking, alcohol consumption, nutrition and drug use. As an example, we focus on the (cleft) palate, for which the zebrafish ethmoid plate is a good model. This review highlights the importance of investigating ExE interactions and discusses the variable effects of exposure to environmental factors on craniofacial development depending on dosage, exposure time and developmental stage. Zebrafish also promise to be a good tool to study novel craniofacial teratogens and toxin mixtures. Lastly, we discuss the handful of studies on gene–alcohol interactions using mutant sensitivity screens and reverse genetic techniques. We expect that studies addressing complex interactions (ExE and GxE) in craniofacial malformations will increase in the coming years. These are likely to uncover currently unknown mechanisms with implications for the prevention of craniofacial malformations. The zebrafish appears to be an excellent complementary model with high translational value to study these complex interactions.

## Introduction

Craniofacial malformations are a heterogenic group of developmental defects of the skull and face, for which no preventive therapies exist. This broad group of over 700 disorders includes debilitating malformations of the skull (craniosynostosis), jaw (micrognathia), face (hemifacial microsomia, deformational plagiocephaly) and teeth (tooth agenesis), (Online Mendelian Inheritance in Man) (OMIM). Heterogeneity is not only a principal feature of craniofacial phenotypes, but also contributes to their etiology, as malformations are often caused by complex interactions of genetic and environmental factors (Gardner et al., 1998; Dixon et al., 2011).

In this review, we focus on clefts of the palate, which oftentimes co-occur with cleft lip. Cleft lip and/or palate (CLP) is the most common congenital craniofacial birth defect. It affects the face uni- or bilaterally, as well as the hard and soft palate (Dixon et al., 2011). Extensive reconstructive surgery and dental treatment are often required until adulthood for the various forms of CLP (Acum et al., 2020; Martin et al., 2020). After surgery, CLP patients may still experience difficulties with speaking and hearing, leading to psychosocial problems (Martin et al., 2020). About 30% of CLP cases arise as part of a syndrome caused by a single genetic mutation. However, the majority of cases are non-syndromic and associated with both genetic (G) and environmental (E) risk factors (Beaty et al., 2011; Dixon et al., 2011; Stuppia et al., 2011).

Candidate genes for CLP are continually identified benefiting from genetic data from individuals, family-and-twin studies, and large-scale GWAS studies (Beaty et al., 2002; Mansilla et al., 2005; Leslie et al., 2015). Often, these genes relate to central regulatory pathways of development including sonic hedgehog (SHH), transforming growth factor (TGF), fibroblast growth factor (FGF), bone morphogenetic protein (BMP), retinoic acid signaling (RA) and Wingless-Int-1 pathway (WNT) signaling (Beaty et al., 2011; Dixon et al., 2011; Reynolds et al., 2019). For example, mutations of the BMP pathway gene *MSX1* and multiple canonical (WNT9B) and non-canonical (WNT5A) *WNT* pathway genes have been associated with non-syndromic CLP (Chiquet et al., 2008). In parallel, environmental factors such as smoking, drinking, drug use, diet and other lifestyle habits during pregnancy have been demonstrated to increase the risk of CLP (van Rooij et al., 2001; DeRoo et al., 2008; Jentink et al., 2010) and other craniofacial malformations (Gardner et al., 1998; Carmichael et al., 2008; Al-Ani et al., 2017). Due to the substantial number of factors involved in craniofacial malformations, the underlying etiology remains largely elusive. Moreover, interactions between genetic and environmental factors (GxE) further complicate the etiology. Since no single animal model can mimic all facets of genetic and environmental influences on craniofacial development in humans, the use of multiple model species is required to elucidate the etiology of craniofacial malformations.

Zebrafish are a promising model to study both genetic and environmental interactions in craniofacial malformations, such as palatal clefts. In the last decade, there has been a rapid increase in zebrafish genetic models for human disease-causing genes including CLP (reviewed by Duncan et al., 2017; Machado and Eames, 2017). Additionally, the growing zebrafish-based research field investigating the role of environmental factors in craniofacial malformations enhances the value of zebrafish in craniofacial research. Zebrafish studies have produced powerful mechanistic insights on both the genetic (variants) and environmental factors which disturb facial development. Up to now, investigations of GxE interactions are published less frequently. However, zebrafish studies in this field are upcoming since there is overwhelming evidence that GxE interactions are the crux of cleft etiology in humans (Wu et al., 2010; Beaty et al., 2011; Dixon et al., 2011; Stuppia et al., 2011).

We have outlined this review as follows: first we discuss zebrafish studies on the effects of maternal smoking, alcohol use, nutrition, drug use and household environmental factors on craniofacial development. We specifically focus on the molecular mechanisms and phenotypic outcomes. Environmental factors with the highest odds ratio (OR) for incidence of craniofacial malformations are discussed first. Secondly, we consider the limited but emerging field of GxE interactions in zebrafish models for craniofacial malformations, and propose strategies for future research.

## Zebrafish as a Craniofacial Model System

The teleost fish, the zebrafish has several advantages over the use of other vertebrate animal models for the study of congenital defects. In particular, zebrafish has a rapid, transparent early development and large brood sizes (Schilling and Kimmel, 1997). They develop a detailed and visible craniofacial skeleton as early as 5 days post fertilization (dpf) (Kimmel et al., 2001). Zebrafish embryos can also be directly exposed to environmental factors via the embryo medium. Zebrafish orthologous genes have been identified for 82% of disease-causing genes in humans as reported in OMIM (Howe et al., 2013). These assets make the zebrafish an attractive model for studying early craniofacial development alongside more traditional animal models such as mouse and chicken (Van Otterloo et al., 2016).

In zebrafish, craniofacial development commences with the migration of cells from the rhombomeres and mesencephalon of the neural crest. These cells migrate in tightly regulated lineages toward predetermined destinations in the head region (Dougherty et al., 2012; Rocha et al., 2020). The first lineage of cranial neural crest cells (CNCC) splits and one part migrates between the developing eyes making up the frontonasal CNCCs. The other part migrates ventrally to populate the first of seven pharyngeal arches (Wada et al., 2005; Swartz et al., 2011; Dougherty et al., 2012). Arches 2–7 are subsequently populated by other lineage of CNCCs, where, upon arrival, all CNCCs surround a mesoderm core (Mork and Crump, 2015). Orchestrated by evolutionary conserved intrinsic and extrinsic signaling, the CNCCs condense and undergo chondrogenic differentiation to form the head cartilages. Comprehensive data and resources on craniofacial development can be found at FaceBase^1^, including “FishFace, an atlas of zebrafish craniofacial development” (Eames et al., 2013).

**FIGURE 1:**
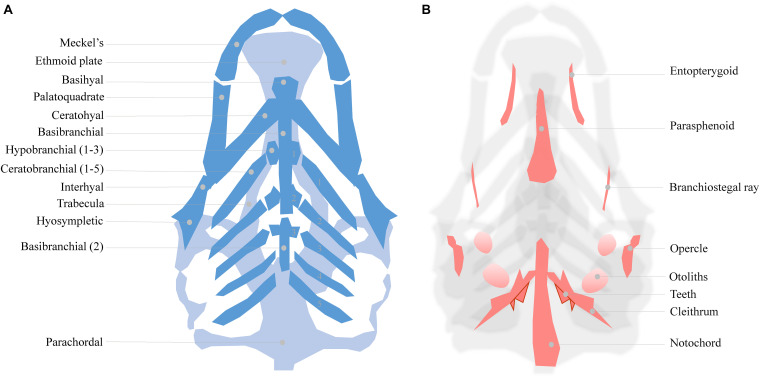
**(A)** Cartilage structures of the zebrafish neuro- and viscerocranium at 5 dpf (light and dark blue, respectively), ventral perspective. A number of these structures is used as a homolog to human skeletal features: Meckel’s cartilage for the lower jaw, the ethmoid plate for the hard palate and the palatoquadrate for the middle ear. **(B)** Intramembranous bones of the 5 dpf zebrafish head, dorsal view.

As mentioned, zebrafish develop a functional craniofacial skeleton within the first 5 days post fertilization (Figure 1). However, the zebrafish craniofacial skeleton is structurally more complex than that of most other vertebrates, and continues to grow throughout the fish’s lifetime (Bruneel and Witten, 2015). The adult zebrafish head contains 73 bones, exceeding the number of bones in the mammalian skull at least three fold (Cubbage and Mabee, 1996). The early cartilage structures consist of only a few chondrocyte and perichondral cell layers (Figure 1A; Kimmel et al., 2001). The first cartilages can be detected as early as 3 dpf, while some intramembranous bone structures also start to form on the cartilage matrix (Aceto et al., 2015). At 5 dpf, a small number of bone structures are already present (detailed in Figure 1B). The cartilages are divided into the dorsally located neurocranial structures (CNCC- and mesoderm- derived) that support the developing brain, and the ventrally located pharyngeal arch-derived structures important for feeding and gill coverage (Kimmel et al., 2010). The cartilages are maintained during early larval stages and are progressively replaced by bone through endochondral ossification (Cubbage and Mabee, 1996; Bruneel and Witten, 2015; Weigele and Franz-Odendaal, 2016). Surprisingly, the zebrafish intramembranous bones are often disregarded in investigations of craniofacial development in the presence of environmental factors, resulting in significant knowledge gaps.

The zebrafish larval head can be used to study various craniofacial malformations. For instance, the cranial sutures of the juvenile (3–12 weeks) zebrafish skull can be manipulated to mimic craniosynostosis, while the Meckel’s cartilage formation can be used study lower jaw defects (Miller et al., 2000; Andreeva et al., 2011; Laue et al., 2011; Cornille et al., 2019). To be concise, we focus on the ethmoid plate and the associated trabecula. These structures are part of the zebrafish neurocranium and are considered homologous to the mammalian palate (Bush and Jiang, 2012).

**FIGURE 2:**
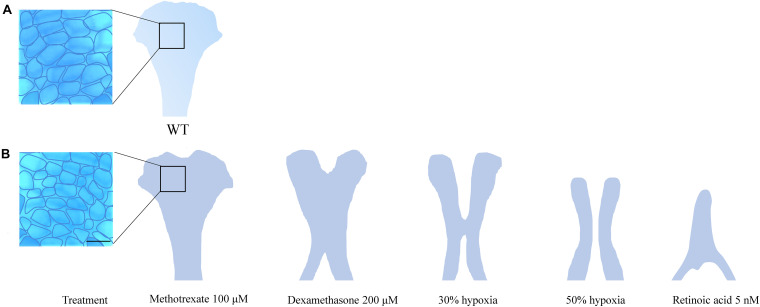
**(A)** Wildtype ethmoid plate with associated stacking of chondrocytes. **(B)** Redrawn examples of ethmoid plate defects observed in zebrafish environmental factor exposure studies (Kuchler et al., 2018; Liu et al., 2020) (Methotrexate 100 μM, Dexamethasone 200 μM, retinoic acid 5 nM and hypoxia). A broad phenotypic range of ethmoid plate defects is observed, indicating that development of the ethmoid plate is affected variously by different compounds. Mild phenotypes include a rough edge to the anterior ethmoid plate, and differential cell morphology (round vs. elongated) and chondrocyte stacking is disordered, drawn from Liu et al. (2020) (scale bar 5 μm). At the other end of the spectrum, phenotypes are observed in which ethmoid plate structures are (partially) missing, indicating effects on migration, differentiation and survival CNCCs. Moreover, some factors affect early as well as late craniofacial development, and various effects can occur though differential exposure times during specific sensitivity windows.

The ethmoid plate and the mammalian hard palate both derive from bilateral streams of anterior maxillary CNCC (Mork and Crump, 2015). In zebrafish, frontonasal CNCCs migrate below the eyes to the midline, while maxillary CNCCs from the first pharyngeal arch assemble laterally to the midline. Then, both lineages fuse to form the ethmoid plate (Dougherty et al., 2013). In humans, mice and, other mammals, the palate develops during gestational weeks 6–12 (humans) and E10–E16 (mice) (Bush and Jiang, 2012). The palate emanates from the outgrowth, vertical-to-horizontal reorientation and fusion of CNCC-derived maxillary prominences (Bush and Jiang, 2012). Mutations in PDGFRA and genes of the SHH pathway lead to inadequate midline fusion resulting in a cleft palate phenotype in mice, humans and zebrafish (Wada et al., 2005; Swartz et al., 2011; Dougherty et al., 2013; McCarthy et al., 2013). These shared disease etiologies confirm that similar molecular mechanisms are involved in the development of the palate and ethmoid plate. A commonly accepted interpretation hereof is that the ethmoid plate is homologous to the mammalian palate (Mork and Crump, 2015). In zebrafish, the cleft phenotype typically features aberrant chondrocyte morphology and stacking in the ethmoid plate. Also, the anterior border of the ethmoid plate can appear dented instead of smooth. More severe ethmoid phenotypes include two anteriorly unconnected outer rods or only a single rod. The spectrum of ethmoid plate disruptions is broad, but some representative examples taken from the literature on environmental factors are represented in Figure 2.

Additional morphological, cellular and molecular parallels between mammals and zebrafish are continually identified in craniofacial research. Virtually every new discovery further supports the translational value of the zebrafish model. We advocate that zebrafish should be used more extensively as an additional tool complementary to established models such as the mouse. In the following sections, we discuss zebrafish models for investigating environmental factors in craniofacial malformations.

## Smoking

In epidemiological studies, maternal smoking before and during early pregnancy is consistently reported as an environmental risk factor for CLP (Stuppia et al., 2011; Sabbagh et al., 2015; Xuan et al., 2016). Despite decades of research, the mechanisms by which smoking affects development are still not fully understood. Since cigarette smoke consists of over 7,000 components, numerous mechanisms are involved (center for disease control) (CDC)^2^. Abundant components of smoke are polycyclic aromatic hydrocarbons (PAHs), carbon monoxide, tar and nicotine (Kennedy et al., 2017). These compounds can exert direct teratogenic effects when transported across the placenta to the amniotic fluid (Liszewski et al., 2012). Cigarette smoke contains inhibitors (nicotine) and inducers (PAH) of the aryl hydrocarbon receptor (AhR) pathway that activates cytochrome P450 1A (CYP1A). During xenobiotic metabolism, reactive products are formed from PAHs, mediated by CYP1A. These products can cause cell damage if they are not rapidly detoxified by metabolite glutathione-S-transferase (GST). Activity of this enzyme can be reduced by smoke itself or by genetic factors (van Rooij et al., 2001). For instance, a maternal GSTT1-null genotype in humans has been shown to increase the risk of clefting in the offspring of smoking mothers (odds ratio 3.2) (van Rooij et al., 2001). Thus, the toxicity of smoking can be further increased by genetic predisposition for suboptimal function of the enzymes for xenobiotic metabolism.

In studies on the effects of maternal smoking on development, zebrafish were traditionally used as a model to examine effects of single abundant compounds of cigarette smoke. Craniofacial malformations are consistently reported in zebrafish studies on smoke compounds such as PAHs, carbon monoxide (hypoxia) and nicotine (Billiard et al., 2006; Parker and Connaughton, 2007; Geier et al., 2018; Kuchler et al., 2018). Specifically, clefts in the ethmoid plate were observed when zebrafish are exposed to hypoxic conditions during early development. These clefts increased in severity as oxygen levels were reduced (Kuchler et al., 2018). Although single-factor investigations are interesting from a fundamental standpoint, they do not provide clinically relevant insights on the effects of maternal smoking on development, because the combined teratogenic effects of smoke compounds may be additive.

In recent years, there has been an increasing amount of literature on the effects of smoke on zebrafish development by exposure to the total particular matter (TPM) of cigarette smoke. TPM exposure lead to craniofacial malformations from above 6 μg/mL, but craniofacial malformations have not yet been evaluated in depth (Ellis et al., 2014; Massarsky et al., 2015; Palpant et al., 2015). Other reported effects of TPM are increased larval mortality, gross morphological deformities and distorted AhR pathway-related enzyme functions (CYP1A and GST). Cross-talk between the AhR pathway and Wnt/β-catenin signaling in zebrafish embryos was also shown. Wnt/β-catenin signaling is a major pathway in craniofacial development (Zhang et al., 2016; Reynolds et al., 2019). Moreover, AhR activation down-regulated *sox9a* during cartilage formation in zebrafish (Xiong et al., 2008). Knockdown of the AhR pathway with simultaneous exposure to TPM reduced morphological defects, however knockdown of CYP1A or CYP1B1 increased defects (Massarsky et al., 2016). This confirms that the toxicity of TPM is partly dependent on the genetic factors controlling xenobiotic metabolism. Predictably, the overall teratogenic effects of TPM on zebrafish larvae were not mimicked by nicotine alone (Massarsky et al., 2015). This exemplifies the importance of evaluating compound interactions.

With the emergence of the e-cigarette, new risk factors for congenital malformations were introduced. Studies show that e-cigarettes are regarded by pregnant women as a safer alternative for tobacco during pregnancy (Wagner et al., 2017). The compounds in e-cigarette liquids differ considerably from regular cigarettes as the main components are nicotine, propylene glycerol and vegetable glycerin (the latter two account for ∼90% of the content) (Schober et al., 2014). Upon partial combustion of propylene glycerol and vegetable glycerin, toxins such as formaldehyde, acrolein, benzene and reactive aldehydes form, that may pose a health risk (Schober et al., 2014). Zebrafish exposed to low levels of propylene glycerol (1.25, 2.5, or 5%, at 6–72 hpf) showed disrupted development, including cardiac defects and morphological malformations (Massarsky et al., 2017). It is concerning that such e-cigarette components are new widespread environmental factors that pregnant women are exposed to.

In e-cigarettes, a vast number of chemicals is also used to add optional flavors. Already, over 7,000 different flavors were on the market in 2014 (Zhu et al., 2014). Flavor ingredients are not listed by manufacturers, possibly because this is not required by law (Tierney et al., 2016). Zebrafish have previously been exposed to fruit flavored e-liquids containing the active compounds ethyl vanillin, maltol and vanillin. It was reported that this caused developmental defects including unspecified craniofacial malformations (Tierney et al., 2016; Holden et al., 2020). The combustion of “berry” and “cream” flavors appeared to cause craniofacial malformations more frequently, but the chemical composition of these (and most other) e-cigarette flavors is unknown. In *Xenopus*, e-cigarette flavors caused craniofacial defects, which were enhanced by co-exposure to nicotine. On its own, nicotine caused only minor malformations (Kennedy et al., 2017). These data confirm interactions between components of e-cigarette liquids and emphasize the need for combined TPM exposure studies. While some e-cigarette flavors have been studied in zebrafish and other animals, considerably more investigations are needed on their interactions and mechanisms of action.

## Alcohol

Fetal alcohol syndrome (FAS) and fetal alcohol spectrum disorders (FASD) are pressing health conditions resulting from alcohol exposure during the first two trimesters of pregnancy. FAS (0.2–1.5 in 1,000 births) describes a multitude of congenital abnormalities of the nervous system, cardiovascular system and face. FASD (1–5 in 100 births) symptoms are less severe but also include central nervous system disorders and craniofacial malformations^3^. The variety in symptoms upon alcohol exposure can be attributed to factors including the timing of alcohol exposure, dosage, metabolism and epigenetics (McCarthy and Eberhart, 2014). Described craniofacial malformations include a flat midface (midface hypoplasia), short nose and smooth philtrum, a thin upper lip, microcephaly, micrognathia and, in 7% of FASD cases, cleft palate (Sampson et al., 1997; Li et al., 2007). Ethanol exposure and FAS/FASD etiology have received ample attention in zebrafish studies and other models, and studies are reviewed frequently (McCarthy and Eberhart, 2014; Facciol et al., 2019; Lovely, 2020).

Ethanol can be found in amniotic fluid after alcohol consumption by the mother and can thus impact the fetus directly. Within 1 h after consumption, fetal alcohol levels are equivalent to those in the maternal circulation (Burd et al., 2007). The known mechanisms of ethanol teratogenicity are many fold. Currently, they are known to include increased cell death, reactive oxygen species, altered growth factor signaling, altered retinoic acid signaling, disrupted cholesterol homeostasis and impaired cell adhesion (Li et al., 2007; Marrs et al., 2010; Soares et al., 2012; Sarmah et al., 2013; Muralidharan et al., 2015; Eason et al., 2017). The zebrafish is an established model to study the effects of ethanol on development. Importantly, the chorion, which surrounds the early zebrafish embryo, appears freely permeable for ethanol. Zebrafish embryonic ethanol levels reach approximately 25–35% of the embryo medium level (Blader and Strahle, 1998; Ali et al., 2011; Fernandes et al., 2019). In zebrafish, both short-term and chronic ethanol exposure increase the incidence of craniofacial malformations.

In search of ethanol sensitivity windows for craniofacial development, zebrafish embryos were exposed to a—rather extreme—regime of 10% ethanol during defined developmental stages (Ali et al., 2011). At 25 and 31 hpf (developmental stages prim-6 and prim-16, respectively) embryos were most susceptible to defects of the branchial arches and Meckel’s cartilage (Ali et al., 2011). Furthermore, in the late blastula and early gastrula stages, embryos appeared to be specifically sensitive for the induction of cyclopia after exposure to 2.4% ethanol (Blader and Strahle, 1998). Upon chronic exposure, distinct craniofacial effects have been observed depending on ethanol dosage. Ethmoid plate development and head width were reduced at concentrations as low as 3 mM (0.01%), which can be reached in women upon drinking only one alcoholic beverage (Carvan et al., 2004; Ferdous et al., 2017). Interestingly, with rising concentrations, a sensitivity shift was reported. At 10 mM ethanol (0.04%), neurocranium structures were more severely affected than structures of the viscerocranium, while at 30 mM (0.13%) the opposite was observed (Carvan et al., 2004). Variations in sensitivity to ethanol exposure between zebrafish strains are also reported. Upon exposure, Ekkwill strain zebrafish presented with a severely affected viscerocranium and increased apoptosis, whereas AB strain zebrafish had affected neurocranial cartilages such as the ethmoid plate. In Tübingen strain zebrafish larvae a high mortality rate was observed, but this strain was less prone to craniofacial defects (Loucks and Carvan, 2004). The strain specific sensitivity to ethanol implicates that predisposing genetic factors and GxE interactions are involved.

Most studies in which zebrafish were exposed to ethanol, used the timeframe between 6 and 24 hpf. This is equivalent to binge drinking (4–5 drinks) throughout the entire first trimester of pregnancy in humans. This poorly resembles a clinically relevant situation and, therefore, Zhang et al. transiently exposed zebrafish to alcohol, during early gastrulation (5.25–6.25 hpf) and neurulation (8–10 and 24–27 hpf) (Zhang et al., 2014). These larvae presented with FASD symptoms, including craniofacial malformations and differential expression of SHH pathway genes. Interaction with SHH signaling seems to be a central mechanism of ethanol teratogenicity (Li et al., 2007; Burton et al., 2017). Upon translation, Shh undergoes posttranslational modifications by cholesterol. This mechanism is crucial for craniofacial development, but ethanol (0.7–7 mM) treatment during gastrulation impaired modification of *shh* by cholesterol (Li et al., 2007). As previously mentioned, SHH pathway gene mutations are also implicated in craniofacial malformations in humans. Later in this review we will discuss evidence of interactions between genetic factors and ethanol, which further complicates the mechanism of action.

## Vitamins and ExE Interactions

Proper development requires a delicate balance of micronutrients. Specifically, derivatives of vitamins A and B are crucial for craniofacial development in both fish and mammals. Zebrafish have been used to study the roles of vitamins A and B during development and in craniofacial malformations. Far less is currently known from the zebrafish model about other essential vitamins and trace elements implicated in craniofacial malformations.

### Vitamin A

Retinoic acid (RA) is a vitamin A-derived retinol and an essential morphogen in embryonic development. Vitamin A is acquired through products such as meat, milk, eggs and carrots (Metzler and Sandell, 2016). Vitamin A deficiency (serum level < 0.70 μmol/L) is a public health concern in countries in Africa and South East Asia, and was associated with craniofacial malformations including CLP (WHO^4^) (Ackermans et al., 2011; Mammadova et al., 2014; Metzler and Sandell, 2016; Williams and Bohnsack, 2019). Supplementation with vitamin A was associated with a lower cleft risk (OR = 0.48) (Johansen et al., 2008). Use of acne medication that contains vitamin A derivatives during pregnancy may result in hypervitaminosis A. This increased the risk of craniofacial malformations including cleft lip, cleft palate, micrognathia and midface hypoplasia (Williams and Bohnsack, 2019).

In mammals, RA precursors are delivered to the fetus by the maternal circulation in the form of retinoids and carotenoids. In zebrafish, RA is synthesized by oxidation of all-trans-retinal derived from the yolk (Metzler and Sandell, 2016). RA subsequently acts through the nuclear retinoic acid receptors. These receptors belong to the steroid/thyroid superfamily (Joore et al., 1994; Metzler and Sandell, 2016). Zebrafish have two (α and γ) RA receptors while mammals have three (α, γ, and β). Retinoic acid receptors are widely expressed throughout the embryo and act by dimerization with retinoid x receptors. Active RA is degraded by CYP26A1 and CYP26B1, and a posterior-anterior increasing gradient of RA is maintained during early development (Williams and Bohnsack, 2019).

RA is a key regulator of Hox-family genes, which are essential for pharyngeal arch development (Williams and Bohnsack, 2019). RA targets the endoderm and ectoderm surrounding the CNCCs, which subsequently signal to CNCCs in the pharyngeal arches. In zebrafish, 1 nM of exogenous RA between 10.5 and 12.5 hpf increased expression of the *hoxa1* and *hoxb2* genes. This resulted in the fusion of structures of the first and second pharyngeal arches, such as fusion of the ceratohyal cartilage with Meckel’s cartilage (Yan et al., 1998). Moreover, transient treatment with 1 μM RA during gastrulation (5.25–10 hpf) resulted in complete loss of expression of the neural crest cell marker *dlx* in the head at 24 hpf, and the absence of craniofacial cartilages. At 0.1 μM RA, *dlx* expression was detected in neurocranial structures, but not in the viscerocranium, suggesting specific sensitivity of these structures (Ellies et al., 1997). Embryos treated with 0.1 μM RA during neural crest migration (12–19 hpf) showed impaired Meckel’s cartilage and palatoquadrate formation, which was also associated with reduced *dlx4* expression. RA treatment caused only mild effects on Meckel’s cartilage upon treatment at 24 hpf, suggesting RA mainly affected CNCC migration (Ellies et al., 1997). In contrast to these findings, at a much lower concentration (5 nM) and using continual exposure between 4 and 96 hpf, exogenous RA had severe effects on the neurocranium. The ethmoid plate was reportedly severely shortened and resembled a single rod. Cells of the ethmoid plate were also disorderly stacked (Liu et al., 2020). After development, RA retains essential functions in regulating osteoblast and osteoclast activity, maintaining bone mineral density, and promoting cell survival (Williams and Bohnsack, 2019). Exogenous RA treatment (0.1 μM) in adult zebrafish during just 5 days resulted in an acute prognathic jaw. Inhibition of RA synthesis resulted in decreased head height (Chawla et al., 2018). Similarly, craniosynostosis phenotypes were observed in CYP26b (hypomorph)-deficient juvenile zebrafish as a result of disrupted RA degradation (Spoorendonk et al., 2008; Laue et al., 2011). These data support that a tight regulation of RA activity is necessary during and after craniofacial development.

Epidemiological studies showed that the risk of FASD and craniofacial malformation were higher in pregnancies with alcohol exposure in low socioeconomic environments. In these environments, maternal malnutrition and vitamin (A) deficiency are more frequent (Jiang et al., 2020). Ethanol competes with retinol for a dehydrogenase that converts retinol into RA and ethanol into acetaldehyde (Marrs et al., 2010). In zebrafish, RA and ethanol appear to exert opposing effects on ethmoid plate development. Zebrafish larvae treated with 100 mM (0.6%) ethanol showed a reduced ethmoid plate width, which was rescued by a low dose (1 nM) of exogenous RA. In the same study, RA addition without alcohol exposure resulted in a wider ethmoid plate (Marrs et al., 2010). Disruption of RA signaling appears to be a distinct mechanism of alcohol teratogenesis and is an example of an ExE interaction.

### Folic Acid

Recommendations are given to pregnant women to commence vitamin B_9_ or folic acid (FA) supplementation prior to conception (WHO)^5^. FA is converted to folate in the body and prevents neural tube defects such as spina bifida, anencephaly and craniorachischisis. FA was also shown to reduce CLP risk in a meta-analysis (Wilson et al., 2015; Millacura et al., 2017). Still, findings are contradictory as specific genetic predisposing factors combined with FA supplementation were reported to be detrimental to development (Marean et al., 2011). FA is required for nucleic acid synthesis and for histone and DNA methylation. Zebrafish embryos starved of folate showed defects early in development, which may be caused by cell cycle delay in the S-phase (Lee et al., 2012). Upon alcohol exposure during pregnancy, inefficient maternal-to-fetal FA transport leads to FA deficiency in the fetus. This is thought to result from reduced expression of folate binding and transport proteins under the influence of ethanol (Hutson et al., 2012). In zebrafish, FA supplementation rescued ethanol-induced craniofacial malformations most effectively when administered during early embryogenesis (6 hpf). Upon addition of FA, apoptosis in the embryos was reduced and *tbx-1* signaling was partially restored (Jiang et al., 2020). Notably, mutations of *TBX-1* have been associated with cleft palate in humans (Herman et al., 2012). The rescue by FA may be mediated by its antioxidant properties and epigenetic restoration of ethanol-induced effects (Muralidharan et al., 2015). The role of FA in birth defect prevention is well-characterized. However, future zebrafish studies may offer more insights into genetic predisposing factors that diminish the efficacy of FA to prevent congenital abnormalities (Marean et al., 2011).

## Pharmaceuticals and Teratogenesis in Zebrafish Larvae

Use of pharmaceuticals during pregnancy can lead to craniofacial malformations. The FDA provides contraindications to pregnant women for the use of drugs associated with teratogenic effects. However, since pregnancies may be unplanned and/or undetected in the first months, it is not always possible to cease use of medication during early pregnancy (Finer and Zolna, 2016). Pharmaceuticals are a prime example of highly controllable environmental factors, as circulating doses in patients are standardized. By using equivalent doses in animal studies, direct comparisons of teratogenic effects can be made between species. In zebrafish, administration of drugs is relatively simple, but care should be taken to minimize effects of embryo medium conditions such as pH, salt concentration and temperature on drug uptake (Weigt et al., 2011).

For example, the widely used anti-epileptic drug and mood stabilizer VPA, prescribed to women of reproductive age, is frequently associated with craniofacial teratogenicity. VPA was reported to cause FVSP, with symptoms such as intellectual disability, facial abnormalities (including CLP) and cardiac defects (Clayton-Smith et al., 2019). VPA acts by inhibition of histone deacetylases, which results in changed expression of genes important for craniofacial development (Phiel et al., 2001; Gurvich et al., 2005). Altered WNT signaling is a known mechanism of VPA teratogenesis. Impaired WNT signaling by genetic or environmental causes is known to result in craniofacial malformations such as CLP in humans. For a comprehensive overview on the involvement of the WNT pathway in craniofacial development we refer to a previous review (Reynolds et al., 2019).

The reported phenotypic effects of VPA on zebrafish vary between studies. However, parallels between human FVSP and zebrafish phenotypes can be drawn. VPA exposure between 4 and 96 hpf (30 μM) resulted in microcephaly, a shortened ethmoid plate with rough anterior edge, and a hypoplastic Meckel’s cartilage with disorganized chondrocyte stacking (Liu et al., 2020). These effects on the ethmoid plate parallel those of the cleft palate phenotype in FVSP. In our lab, early (1–13 hpf) and late (25–37 hpf) embryonic VPA exposure (50 and 100 μM VPA) resulted in a shortened head, reduced bone formation and smaller ceratohyals. Wnt marker *axin2* was downregulated, while the Wnt inhibitor *dkk1b* was upregulated at 5 dpf. This may account for the decreased ossification, as Wnt is essential in initiating ossification (Gebuijs et al., 2020). Martinez and colleagues reported on a dramatic decrease of survival and lack of development of the craniofacial cartilages in embryos exposed to 25, 50, and 100 μM at 4–96 hpf. The long exposure window used in this study may account for the phenotypic discrepancy with the results obtained in our lab.

In addition to VPA, many other drugs may affect craniofacial development. Obviously, the effects depend on the dose as well as on the exposure duration or corresponding developmental stage. A succinct, tabular overview of effects and mechanisms of selected teratogens from major drug classes is provided in Table 1. We systematically compared the effects reported in zebrafish with phenotypes described in humans (or if not available, in mouse models). Zebrafish models largely mimicked the phenotypic effects that were observed in mammals, and especially humans, in six out of eight selected teratogens from various drug classes. The target mechanisms implicated in craniofacial development largely varied, but in three cases a direct etiological link with major developmental pathways could be made. Other mechanisms involved direct effects on transcription or DNA synthesis. The translational value as well as the predictive value of the zebrafish model for the study of craniofacial malformations caused by teratogenic pharmaceuticals is demonstrated.

**TABLE 1:** Reported effects of pharmaceuticals at various concentrations and exposure times on zebrafish craniofacial development and parallel craniofacial findings in humans or, if not available, in mice.

Compound	Concentration	Exposure time	Effects in zebrafish	References	Target mechanisms in craniofacial development	Effects on mammals
Phenytoin (Anti-epileptic)	200 μM	4–96 hpf	Irregular **anterior ethmoid** border and rounder cells, **shortened head**	Liu et al., 2020	Upregulated retinoic acid receptorsand growth factors IGF-2, TGFa, and TGFb1, (Gelineau-van Waes et al., 1999)	Causes fetal Hydantoinsyndrome with **microcephaly** cleft lip and/or **cleft palate** in humans (Gelineau-van Waes et al., 1999). In mice cleft lip and **palate** (Sulik et al., 1979; Mao and Tang, 2010)
Warfarin (Anticoagulant)	52.4 μM	2.5–72 hpf	Severe head malformations (unspecified), **eye and otolith defects**	Weigt et al., 2012	Upregulated Bmp antagonist *tsku*. Inhibited glutamyl to γ-carboxyglutamyl transition of vitamin K-dependent proteins such as osteocalcin and periostin (Hanumanthaiah et al., 2001; Fernandez et al., 2014)	Craniofacial malformations of the nose and airways, **eye/ear defects** and cleft lip in humans when taken during the first trimester of gestation (Hou, 2004)
	60 μM	4–96 hpf	Shortened head structures, a dent in the anterior ethmoid plate, which also showed altered cell morphology and disorganized cell stacking	Liu et al., 2020		
Methotrexate (Immunosuppressor)	100–200 μM	4–96 hpf	**Size reduction of the neurocranium, micro clefts in the anterior ethmoid plate.** Cells that make up the ethmoid plate appeared disorganized	Lee et al., 2013; Liu et al., 2020	Folic acid antagonist which inhibits DNA synthesis and cell proliferation by competitively inhibiting dihydrofolate reductase (Lee et al., 2012)	Craniosynostosis and **microcephaly and incomplete cleft palate** in humans (Granzow et al., 2003; Zarella et al., 2016)
Acetaminophen (Paracetamol)	2.5–13.4 mM	0–120/72–120 hpf	Palatoquadral length, ceratohyal length and head size reduced	Cedron et al., 2020	Activation cytochrome P450 and increased apoptosis (Cedron et al., 2020)	No affected phenotypes reported
Cyclosporin A (Immunmodulator)	10 μM	8–120 hpf	Hypoplasia of the ceratohyoids and ceratobranchials, and **reduced cartilage formation** in of the viscerocranium	Seda et al., 2019	Inhibitor of calcineurin, which dephosphorylates transcription factors Nuclear factor of activated T-cells, upon which these enter the cell nucleus and drive transcription (Winslow et al., 2006)	Cleft palate in mice (Gasser et al., 1992). Nfatc1-deficient mice exhibit reduced **reduced formation of mineralized bone** resulting in wider cranial sutures (Winslow et al., 2006)
Hydroxyurea (Sickle-cell anemia and psoriasis)	1 mM	4–96 hpf	Shortening of the head resembling micrognathia and **microcephaly**	Liu et al., 2020	Largely unknown. Ribonucleotide reductase inhibitor, by which DNA synthesis is inhibited (Woo et al., 2004)	In mice **microcephaly**, and hydrocephalus (Woo et al., 2004)
Leflunomide (Anit-rheumatic drug)	10 μM	8–120 hpf	Craniofacial cartilages did not form	Seda et al., 2019	Inhibitor of Dihydroorotate dehydrogenase (DHODH) (Pinto and Dougados, 2006)	Miller syndrome caused by DHODH gene defects micrognathia cleft palate cleft lip in humans (Ng et al., 2010)
	10 μM	8–24 hpf	Increase in the ceratohyal angle	Seda et al., 2019		
Dexamethasone (Corticosteroid)	200 μM	4–96 hpf	Shortening of the head region and **rough edge to the anterior ethmoid plate** and round, small chondrocytes	Liu et al., 2020	Increase of matrix metalloproteinase 2 and 9 expression and activity, trough the glucocorticoid receptor. Degradation of extracellular matrix components (Hillegass et al., 2008)	**Cleft palate** in mice via inhibiting Wnt/β-catenin signaling (Hu et al., 2013)
	254.81 μM	3–72/96 hpf	Shortened Meckel’s cartilage	Hillegass et al., 2008		

*Phenotypic parallels in bold.*

## Estrogens and Craniofacial Malformations

Endocrine disruption in the embryo can occur by the intake of estrogen-resembling chemicals that disrupt estrogen signaling, such as oral contraceptives. Although no effects on craniofacial development have been reported on continued contraceptive pill use during pregnancy, abundant sources of exogenous estrogens in the (house/work) environment are present, for instance in food (Paterni et al., 2017). Estrogen-like compounds in household items can affect craniofacial development and analyzing such exposures in patients is complex. Zebrafish have been used to study the effects of supraphysiological doses of exogenous (xeno) estrogens on craniofacial development (Cohen et al., 2014).

Estrogens are crucial for development and act through nuclear estrogen receptors (ER) alpha and beta, that (hetero and homo) dimerize to form DNA binding domains (Tanko et al., 2008). Additionally, estrogens act by binding to transmembrane G-protein GPR-30, which induces signaling cascades via phospholipase C activation (Tanko et al., 2008). Endogenously, estrogens are synthesized from androgens by aromatase. Estrogens are pivotal for the maintenance of bone and cartilage. This is illustrated by the presence of ER on mesenchymal stem cells during chondrogenesis and the production of estrogens by chondrocytes to stimulate their proliferation (Chagin et al., 2006; Fushimi et al., 2009; Gao et al., 2013).

Zebrafish larvae exposed to exogenous 17ß-estradiol (E2), show dose and developmental stage-dependent craniofacial malformations with enhanced sensitivity during early chondrogenesis (1–2 dpf) (Fushimi et al., 2009). At E2 concentrations of 3–5 μM, craniofacial cartilages appeared flattened (Cohen et al., 2014). The angles of the Meckel’s and ceratohyal cartilages were widened (Cohen et al., 2014). Below 3 μM, the length and width of craniofacial structures decreased with increasing concentrations (Cohen et al., 2014). Notably, the inhibition of estrogen biosynthesis with aromatase inhibitors phenocopied these results, exemplifying the requirement for tightly controlled estrogen biosynthesis during chondrogenesis (Cohen et al., 2014).

E2 effects on craniofacial development have been partially attributed to indirect inhibition of Shh signaling through estrogen receptor signaling (Fushimi et al., 2009). E2 impaired the migration of CNCCs into the median ethmoid plate, which resulted in a cleft phenotype (Dougherty et al., 2012). In addition to effects on Shh signaling, E2 resulted in differential expression of other skeletogenic pathway genes in an elaborate investigation of estrogen (2–5 μM) effects on zebrafish larval heads (3–7 dpf). Reduced expression of *bmp2a* was reported at 3 dpf. *Bmp2a* has a crucial function in cartilage and bone formation. Also, the osteoclast differentiation factor *rankl* was down-regulated at 3 and 4 dpf. Further, the inhibitor of the WNT pathway *sfrp1a* was down-regulated at 3 and 4 dpf, and the expression of hedgehog receptors *ptch1* and *ptch2* as well as the retinoic acid receptor *rarab* was decreased throughout development (3–7 dpf). Interestingly, many genes in these pathways were affected differently at 2 and 5 μM exposure, while the effects of 2 μM were often more dramatic (Pashay Ahi et al., 2016). When using much lower concentrations of E2 (0.8 μM) cartilage degradation was observed at 4 dpf and collagen genes were down-regulated at 6 and 7 dpf (He et al., 2018). In addition, E2 was reported to upregulate Wnt pathways genes such as *wdr62* (implicated in microcephaly: OMIM 613583) and *wnt11* which has been associated with non-syndromic CLP. The same study reported upregulation of *fstl1a*, a regulator of Bmp signaling, and upregulation of the Bmp inhibitor *nog1*, which is implicated in cleft lip etiology. Moreover, Tgfb receptor 1 expression was downregulated. Variations in this gene cause Loey-Dietz syndrome, which includes craniofacial malformations (He et al., 2018). Overall, exogenous E2 had effects on expression of multiple genes that are involved in craniofacial development.

Recently, a number of zebrafish studies have focused on the effects of bisphenols. Bisphenol A (BPA) is an environmental estrogen-like agent. BPA is found in humans in amniotic fluid, placental tissue and umbilical cord blood (Lee et al., 2018). Pregnant women are exposed to BPA via food packaging, dental fillings and inhalation of house dust (Rudel et al., 2003; Vandenberg et al., 2010; Lofroth et al., 2019). BPA binds to the ER, but it can also bind to the thyroid hormone receptor and to the estrogen-related receptor ERRγ (Saili et al., 2013). BPA is less potent than E2: similar morphological defects were found in zebrafish with 15 μM E2 and 80 μM BPA upon exposure between 8 and 120 hpf (Saili et al., 2013). It should be noted that the concentrations used in these studies exceed environmental concentrations. In zebrafish larvae, BPA induced craniofacial malformations by disrupting chondrocyte organization in the pharyngeal structures and inducing apoptosis (Huang et al., 2020). The BPA-based polymer Bis-GMA used in dental fillers decreased palatoquadral length in zebrafish larvae at 10 nM, while the palatoquadral-ceratohyal angle was decreased at 1 μM (Kramer et al., 2016). It was hypothesized that these effects are caused by ER-mediated altered expression of SHH and BMP pathway genes. Because of emerging evidence of health problems related to bisphenol, the use of BPA was restricted by the European Union.

Upon the reduction of BPA use, products containing alternative bisphenols including BPF, BPS and BPAF have spiked. Because of structural similarities to BPA, concerns also exist about the estrogenic properties of these compounds. BPF, BPS and BPAF are also identified in food products and house dust. In zebrafish, BPF reportedly resulted in severe craniofacial abnormalities at 20 mg/L. BPS caused malformations above 200 mg/L in zebrafish, while BPAF produced severe cardiac defects at 1 mg/L (Moreman et al., 2017). Indeed, the concentrations used in this study also exceed environmental BPA concentrations. Future studies on bisphenols with detailed description of phenotypes and realistic exposure concentrations will provide more insight into the mode of action of various bisphenols on craniofacial development.

## Exe Interactions in High-Throughput Screenings

Craniofacial malformations often serve as outcome measures in zebrafish toxicological investigations. This is because the development of craniofacial structures is highly sensitive to perturbation. Many of the environmental factors discussed in this review have also been investigated in toxicological studies, but these studies lacked specific phenotypic and/or mechanistic information. In general, craniofacial abnormalities were regularly reported in compound studies in zebrafish, but without specifically analyzing the affected structures (Weigt et al., 2012; Ellis et al., 2014; Massarsky et al., 2015). Although purely toxicological investigations are beyond the scope of this review, the methodologies employed in this field can also be used in the study of craniofacial malformations.

For example, investigations of the cumulative effects of teratogens (i.e., ExE interactions) within the zebrafish model are increasing in number. Methods to assess the combinatorial effects of two compounds, synergistic or antagonistic, are currently being developed for toxins with effects on skeletal development (Staal et al., 2018; Heusinkveld et al., 2020; Zoupa et al., 2020). For instance, triazoles (fungicides) cause craniofacial malformations through inhibiting RA degradation by CYP26. These toxins with well-known mechanism of action have been used in co-exposure experiments with other agricultural fungicides that target different processes in craniofacial development. Thereby it was shown that binary mixture effects can be predicted using dose addition. This refers to adding up the toxicological effects of two chemicals to predict their effect in a mixture (Zoupa et al., 2020). This method may be helpful in further studies on multifactorial environmental exposures such as TPM of (e-cigarette) smoke.

**FIGURE 3:**
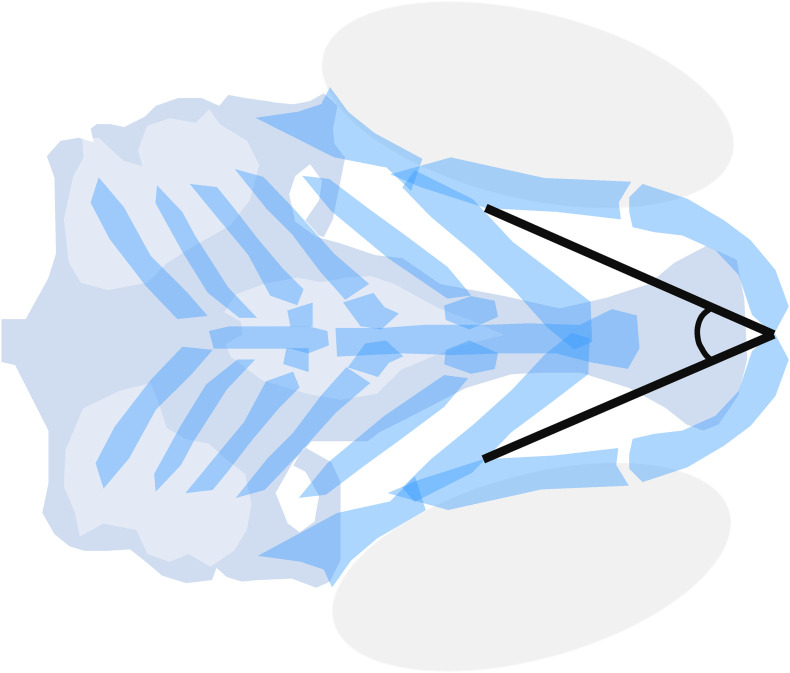
Meckel’s-palatoquadrate (M-PQ) angle is proposed as a reliable high-throughput standard parameter to assess craniofacial outcomes after single or mixed compound exposures. The measurements can be easily obtained by imaging of cartilage stained larvae and proved to be informative on a broad spectrum of craniofacial malformations. The M-PQ angle is especially affected in craniofacial malformations such as microcephaly and micrognathia.

To unify efforts in craniofacial toxicity screenings, standard outcome measures were optimized by Staal et al. (2018). The Meckel’s-palatoquadrate (M-PQ) angle (Figure 3) offered a limited variability, proved applicable over wide concentration ranges, and was suitable to assess the effects of chemical mixtures (Cohen et al., 2014; Staal et al., 2018; Zoupa et al., 2020). The M-PQ angle can be translated as a measure for head and jaw deformations, which is affected in craniofacial malformations such as microcephaly and micrognathia. This relatively simple to obtain parameter is especially suitable for high-throughput screenings. It was also proposed to use in further studies in order to enhance inter-study data comparisons. Similarly, options for high-throughput phenotyping of craniofacial mutants by microcomputed tomography (microCT) are rapidly expanding, benefiting from improved resolution and software tools (Pardo-Martin et al., 2013; Charles et al., 2017). In pathophysiological investigations of craniofacial malformations using zebrafish, the techniques discussed above may be used to systematically assess, or even predict, the teratogenesis of novel environmental factors.

## Gene–Environment Interactions

It has become increasingly evident that the effects of environmental exposures during development may be different in individuals with different genotypes. Such interactions of genetic and environmental factors are poorly understood up to now (Lovely et al., 2017). A limited number of zebrafish studies have addressed the interactions between genetic risk factors and environmental factors in the etiology of craniofacial malformations. These studies focused on ethanol sensitivity of zebrafish mutants in FASD research. Screens of n-ethyl-n-nitrosourea (ENU)-induced mutants and unbiased screens for new candidate genes have been published. Mutants were generally exposed to 1% ethanol during development. This results in tissue levels between 41 and 51 mM, which correlates to blood-alcohol levels after binge drinking events in humans (Zhang et al., 2014). In search of GxE interactions, McCarthy et al. (2013) exposed *Pdgfra* zebrafish mutants to 1% ethanol. Loss of PDGFRA in humans has been associated with cleft palate, indicating a direct parallel with zebrafish studies (Rattanasopha et al., 2012). *Pdgfra^+/–^* zebrafish mutants only developed craniofacial malformations in the presence of ethanol (10–120 hpf) including clefts of the ethmoid plate and breaks in the trabeculae. Moreover, hypoplasia of Meckel’s cartilage, the palatoquadrate and the hyosympletics were observed. *Pdgfra^–/–^* mutants showed enhanced sensitivity to ethanol as the ethmoid plate did not form at all, while the sensitivity window was determined at 10–24 hpf. Wildtype embryos exposed to ethanol developed mostly normal in this study with palatal defects only in less than 20% of the larvae. The protective effect of intact *pdgfra* against ethanol teratogenesis was shown to be PI3K/mTOR-mediated (McCarthy et al., 2013). This type of in-depth examinations of the mechanisms of action are needed to extrapolate models of gene–environment interactions in craniofacial disorders to clinical use.

Swartz et al. found that mutations of *foxi1, hinfp, mars, plk1* and *vangl2* also showed increased craniofacial malformations upon exposure to 1% ethanol compared to wildtypes (Swartz et al., 2014). In particular, ethmoid plate defects were observed in both *mars* and *vangl2* mutants exposed to ethanol. *Mars* is crucial for protein synthesis as it is involved in tRNA amino acylation. In *mars* mutants a gap in the posterior ethmoid palate was observed upon ethanol exposure. *Vangl2* is a regulator of the planar cell polarity Wnt pathway, and is expressed in the pharyngeal arches. In zebrafish mutants for this gene a narrow, single rod replacing the ethmoid plate was reported as a result of ethanol exposure (Swartz et al., 2014). In humans, VANGL2 is implicated in neural tube defects (Kibar et al., 2011). However, based on the zebrafish study, VANGL2 variations may also be a predisposing factor for cleft palate in case of alcohol use during pregnancy.

The screening of a library of unmapped (unknown alleles) ENU mutants recently identified six new ethanol-sensitive zebrafish mutants (Swartz et al., 2020). The observed malformations in these mutants were highly diverse, which suggests a variety of teratogenic effects of ethanol. One of the mutants was mapped by whole genome sequencing and was validated by targeting the same allele using CRISPR/Cas9 mutagenesis (Swartz et al., 2020). Other mutants of this study remain unmapped, but they seem to be new candidate genes for craniofacial malformations as the specific phenotypes were not previously observed (Swartz et al., 2020). This is a prominent example of why both candidate-based approaches and mutant-sensitivity screens are needed in research on GxE interactions. Candidate gene-driven research is focused whereas sensitivity screenings can lead to the discovery of new candidate genes.

The discovery of ethanol-sensitive gene variants in zebrafish shows that the effects of ethanol on craniofacial development may depend on the genetic background. It is highly conceivable this also applies to the teratogenic effect of other compounds. Yet, mechanistic insights into other GxE interactions are still scarce. CLP risks have been associated with genetic sensitivity for specific environmental factors. For instance, TGFA variations may interact with smoking and vitamin deficiency, TGFB3 variations with smoking and alcohol consumption, MTHFR variations with folate intake, RARA with vitamin A intake, and finally GSTT1 and CYP1A variations also with smoking (Stuppia et al., 2011). Zebrafish models are highly suitable to investigate the molecular mechanisms in these gene–environment interactions.

It is likely that zebrafish craniofacial mutants will be used increasingly to analyze the mechanisms of GxE interactions in the coming years. A number of strategies may be appropriate to approach this. The eminent availability of zebrafish mutants from the zebrafish mutation project^6^ and other sources, will facilitate high-throughput investigations of GxE interactions on craniofacial development. These efforts can be further optimized by using the broad range of available reporter lines for bone and cartilage formation (Hammond and Moro, 2012). Furthermore, advanced genome editing offers unprecedented opportunities for the investigation of GxE interactions of candidate genes. For instance, the first successful attempt to create loss-of-function alleles for all genes on zebrafish chromosome 1 was recently published, illustrating the astonishing strength of CRISPR/Cas9 techniques (Sun et al., 2019). Systematic gene editing to create zebrafish variants of those observed in human patients could be achieved in the future in view of the increasing success rates of knock-in zebrafish models. These models can be used for highly specific GxE interaction analyses, which may provide predictive data to clinicians.

## Concluding Notes

Craniofacial malformations are notorious for their multifactorial etiology including many genetic and environmental factors. CLP and other congenital traits in which GxE interactions play a prominent role, present a problem for the development of adequate preventive measures. Here, evidence from zebrafish studies on the phenotypic outcomes and mechanisms of action of a number of important environmental factors that pregnant women may encounter in daily life is reviewed. It stands out that the mechanisms of action for most of these factors remain incompletely understood, while the phenotypic descriptions vary widely in the amount of details given.

**FIGURE 4:**
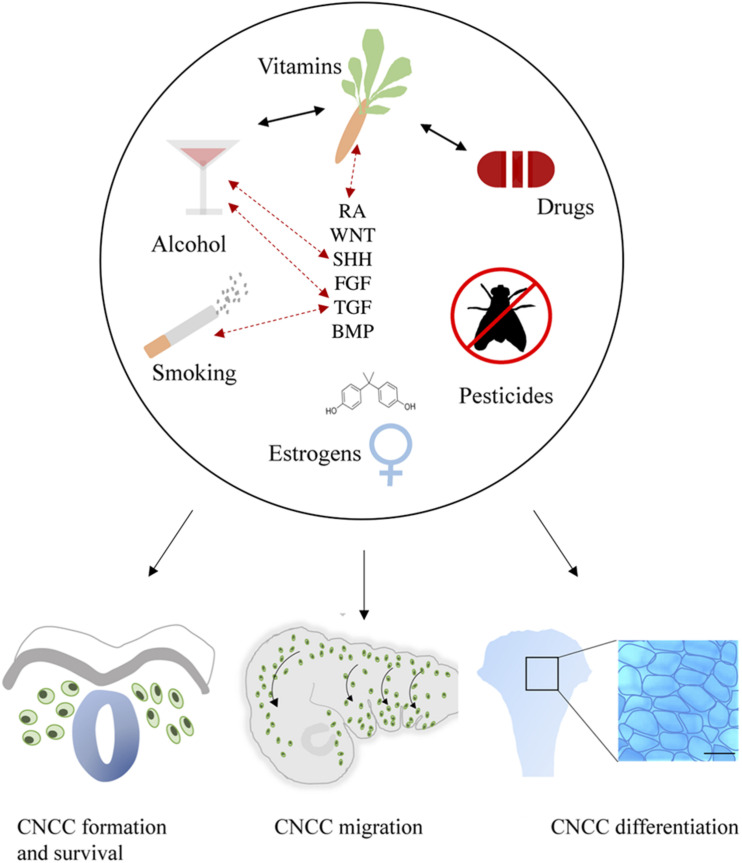
General summary showing the environmental factors smoking, alcohol use, vitamin imbalance, drug use, (xeno) estrogens and pesticides. These factors (represented in the upper part of the figure) differentially affect critical developmental processes such as the formation, survival, delamination, migration, condensation, and differentiation of CNCCs (represented in the lower part of the figure). Evidence of interactions between environmental factors that result in craniofacial malformations has been reported as well, these are indicated by black arrows. Exposure to environmental factors often results in aberrant signaling of essential pathways in craniofacial development including SHH, TGF, FGF, BMP, RA, and WNT. Moreover, gene mutations in these pathways can also interact with environmental factors, complicating the etiology. Known GxE interactions are indicated in this figure with red dotted arrows.

Craniofacial development is a highly sensitive process, which is regulated by major signaling pathways including WNT, BMP, FGF, SHH, and RA. This review substantiates that environmental factors may directly or indirectly affect these pathways and thereby disrupt CNCC formation, survival, migration and differentiation (Figure 4). It is not beyond imagination that multiple environmental factors can be present simultaneously during early pregnancy. Therefore, studies into ExE interactions are highly important.

Zebrafish prove to be a valuable translational model for a wide range of craniofacial malformations, and are also highly suited for identifying the mechanisms of action associated with environmental factors and GxE interactions. Moreover, zebrafish research will boost the identification and health risk assessment of new teratogens.

Although many health recommendations to prevent craniofacial and other congenital malformations have already been implemented by healthcare professionals for years, more research should be done to obtain a more extensive picture of the risks. After all, in the field of environmental exposures there is much to gain as—in theory—all of these factors can be eliminated if public health awareness is increased and risks are adequately identified. A better understanding of environmental teratogens and genetic risk factors will allow for routine screening and a personalized approach in the future. Evidence from zebrafish studies will help to translate preclinical data to practice and will support health recommendations to pregnant women to decrease the risks of CLP and other craniofacial malformations.

## Author Contributions

SR wrote the manuscript. JM, FW, and JV conceptualized and revised the manuscript. All authors contributed to the article and approved the submitted version.

## Conflict of Interest

The authors declare that the research was conducted in the absence of any commercial or financial relationships that could be construed as a potential conflict of interest.

## Footnotes

1 www.facebase.org/

2 https://www.cdc.gov/biomonitoring/tobacco.html

3 www.cdc.gov/ncbddd/fasd/data.html

4 www.who.int/elena/titles/vitamina_pregnancy/en/

5 www.who.int/elena/titles/guidance_summaries/daily_iron_pregnancy/en/

6 www.sanger.ac.uk/resources/zebrafish/zmp/

## Abbreviations

AhR: Aryl hydrocarbon receptor
BMP: Bone morphogenetic protein
BPA/AF/S/F: Bisphenol A/AF/S/F
CDC: Center for disease control
CLP: Cleft lip and/or palate
CNCC: Cranial neural crest cells
CYP1A: Cytochrome P450 1A
DHODH: Dihydroorotate dehydrogenase
dpf: Days post fertilization
E2: 17ß-estradiol
ENU: N-ethyl -n-nitrosourea
ER: Estrogen receptors
FA: Follic acid
FAS: Fetal alcohol syndrome
FASD: Fetal alcohol spectrum disorders
FDA: Food and drug administration
FGF: Fibroblast growth factor
FVSP: Fetal valproate spectrum disorder
GST: Glutathione-S-transferase
GWAS: Genome wide association studies
hpf: Hours post fertilization
M-PQ: Meckel’s-palatoquadrate
OMIM: Online Mendelian Inheritance in Man
OR: Odds ratio
PAH: Polycyclic aromatic hydrocarbons
RA: Retinoic acid signaling
SHH: Sonic hedgehog
TGF: Transforming growth factor
TPM: Total particular matter
VPA: Valproic acid
WHO: World health organization
WNT: Wingless-Int-1 pathway.
