# Corrigendum: Zebrafish Models of Craniofacial Malformations: Interactions of Environmental Factors

**DOI:** 10.3389/fcell.2021.650948

**Published:** 2021-06-24

**Authors:** S. T. Raterman, J. R. Metz, Frank A. D. T. G. Wagener, Johannes W. Von den Hoff

**Affiliations:** ^1^Radboud Institute of Molecular Life Sciences, Nijmegen, Netherlands; ^2^Department of Dentistry-Orthodontics and Craniofacial Biology, Radboud University Medical Center, Nijmegen, Netherlands; ^3^Department of Animal Ecology and Physiology, Institute for Water and Wetland Research, Radboud University, Nijmegen, Netherlands

**Keywords:** zebrafish, craniofacial malformations, neural crest cells, environment, gene, interaction

In the original article, there was an error. The error concerned reported exposure percentages.

A correction has been made to reported percentages in **Alcohol**, paragraph three:

“In search of ethanol sensitivity windows for craniofacial development, zebrafish embryos were exposed to a—rather extreme—regime of 10% ethanol during defined developmental stages (Ali et al., 2011). At 25 and 31 hpf (developmental stages prim-6 and prim-16, respectively) embryos were most susceptible to defects of the branchial arches and Meckel's cartilage (Ali et al., 2011). Furthermore, in the late blastula and early gastrula stages, embryos appeared to be specifically sensitive for the induction of cyclopia after exposure to 2.4% ethanol (Blader and Strahle, 1998). Upon chronic exposure, distinct craniofacial effects have been observed depending on ethanol dosage. Ethmoid plate development and head width were reduced at concentrations as low as 3 mM (0.01%), which can be reached in women upon drinking only one alcoholic beverage (Carvan et al., 2004; Ferdous et al., 2017). Interestingly, with rising concentrations, a sensitivity shift was reported. At 10 mM ethanol (0.04%), neurocranium structures were more severely affected than structures of the viscerocranium, while at 30 mM (0.13%) the opposite was observed (Carvan et al., 2004). Variations in sensitivity to ethanol exposure between zebrafish strains are also reported. Upon exposure, Ekkwill strain zebrafish presented with a severely affected viscerocranium and increased apoptosis, whereas AB strain zebrafish had affected neurocranial cartilages such as the ethmoid plate. In Tübingen strain zebrafish larvae a high mortality rate was observed, but this strain was less prone to craniofacial defects (Loucks and Carvan, 2004). The strain specific sensitivity to ethanol implicates that predisposing genetic factors and GxE interactions are involved.”

A correction has been made to reported percentages in **Vitamins and ExE Interactions, Vitamin A**, paragraph four:

“Epidemiological studies showed that the risk of FASD and craniofacial malformation were higher in pregnancies with alcohol exposure in low socioeconomic environments. In these environments, maternal malnutrition and vitamin (A) deficiency are more frequent (Jiang et al., 2020). Ethanol competes with retinol for a dehydrogenase that converts retinol into RA and ethanol into acetaldehyde (Marrs et al., 2010). In zebrafish, RA and ethanol appear to exert opposing effects on ethmoid plate development. Zebrafish larvae treated with 100 mM (0.6%) ethanol showed a reduced ethmoid plate width, which was rescued by a low dose (1 nM) of exogenous RA. In the same study, RA addition without alcohol exposure resulted in a wider ethmoid plate (Marrs et al., 2010). Disruption of RA signaling appears to be a distinct mechanism of alcohol teratogenesis and is an example of an ExE interaction.”

The authors apologize for these errors and state that this does not change the scientific conclusions of the article in any way. The original article has been updated.
